# The effects of a low carbohydrate diet on erectile function and serum testosterone levels in hypogonadal men with metabolic syndrome: a randomized clinical trial

**DOI:** 10.1186/s12902-023-01278-6

**Published:** 2023-02-02

**Authors:** Caio da Silva Schmitt, Carla Martins da Costa, José Carlos Stumpf Souto, Lorenzo Miron Chiogna, Zilda Elizabeth de Albuquerque Santos, Ernani Luis Rhoden, Brasil Silva Neto

**Affiliations:** 1 Department of Urology, Hospital Moinhos de Vento, Porto Alegre, RS Brazil; 2 Post Graduate Program in Medicine: Surgical Sciences, School of Medicine, Universidade Federal Do Rio Grande Do Sul, Porto Alegre, RS Brazil; 3 Department of Nutrition, Universidade Federal Do Rio Grande Do Sul, Porto Alegre, RS Brazil; 4 Department of Surgery, School of Medicine, Universidade Federal de Ciencias da Saude de Porto Alegre, Porto Alegre, Brazil; 5 Department of Surgery, Hospital de Clínicas de Porto Alegre, Universidade Federal Do Rio Grande Do Sul, Porto Alegre, RS Brazil

**Keywords:** Hypogonadism, Metabolic syndrome, Low carbohydrate diet, Erectile dysfunction, Obesity

## Abstract

**Purpose:**

Metabolic syndrome is a risk factor for several diseases. The relationship between metabolic syndrome and hypogonadism is well known. Our objetive is to assess whether a low carbohydrate diet can increase total serum testosterone and improve erectile function in hypogonadal men with metabolic syndrome.

**Methods:**

An open label randomized clinical trial was conducted comparing a low carbohydrate diet and controls, during three months, in hypogonadal men with metabolic syndrome. Anthropometric measurements were evaluated as well as total serum testosterone levels, and symptoms of hypogonadism, using the ADAM and AMS scores, and sexual function using IIEF-5 score.

**Results:**

Eighteen men were evaluated. Anthropometric measures were improved only in low carbohydrate diet group. The intervention group also had a statistically increase in IIEF-5 score and a significant reduction in AMS and ADAM scores (*p* < 0.001). The increase in serum total testosterone levels was statistically significant in the low carbohydrate group compared to the control group as well as calculated free testosterone (*p* < 0.001).

**Conclusions:**

Low carbohydrate diet may increase serum levels of testosterone and improve erectile function in hypogonadal men with metabolic syndrome. However, larger studies are necessary to strongly prove the effectiveness of low carbohydrate diet in treating male hypogonadism.

## Introduction

Metabolic syndrome has been widely studied in recent years, mainly due to its role as a risk factor for several diseases. Overweight or obesity, which is one component of metabolic syndrome, has a high prevalence and is considered a public health problem. Brazilian data show that the obesity rate in individuals aged ≥ 20 years more than doubled (from 12.2 to 26.8%) between 2003 and 2019 and, during this period, male obesity rose from 9.6% to 22.8% [1]. The 2012 National Health and Nutrition Examination Survey estimated the prevalence of obesity in the US population at 35% [2].

Testosterone deficiency, also called hypogonadism, is also a prevalent condition, affecting around 5 million Americans [3]. Hypogonadism is defined both biochemically and clinically: total serum testosterone < 300 ng/dL and clinical manifestations such as cognitive deficit, decreased lean mass, lower bone mineral density, erectile dysfunction, decreased libido, etc. [3]. Metabolic syndrome, diabetes mellitus and hypogonadism can negatively influence male health and some authors have even proposed that hypogonadism should be included as a criteria for metabolic syndrome [4, 5]. Some studies have shown that low serum testosterone levels are associated with increased insulin resistance, including an estimated 50% prevalence of hypogonadism in diabetic men [6]. Insulin resistance, in turn, is a risk factor for metabolic syndrome and cardiovascular diseases [7].

Metabolic syndrome and all of its risk factors (eg, cardiovascular disease, diabetes mellitus and obesity) are associated with erectile dysfunction, raising the hypothesis that correcting these factors could improve the sexuality of men who suffer from this condition [8]. In addition, some studies not only corroborated these findings, but also demonstrated that a low-carbohydrate diet was superior regarding weight loss and reducing systolic blood pressure, increasing HDL and reducing triglyceride levels, proving to be more effective in reducing established cardiovascular risk factors [9, 10].

We found no studies reporting specific effects of a low-carbohydrate diet on male hypogonadism. Therefore, we performed a study to verify the effects of this type of diet in men with metabolic syndrome, mainly focusing on serum testosterone level and erectile function, due to the possible correlation between them after an extensive literature review.

## Methods

This open label randomized clinical trial was conducted at the Hospital de Clínicas de Porto Alegre (HCPA), Brazil, allocating participants to a low-carbohydrate diet or to their usual diet as a control group. The Random App from Apple Store was used for randomization. The participants, recruited through consecutive sampling, included men > 18 years of age diagnosed with metabolic syndrome and subnormal testosterone levels. The exclusion criteria were cardiomyopathy, heart failure, previous or current neoplastic disease, uncontrolled liver disease, current treatment for erectile dysfunction, used drugs known to interfere with testosterone levels, previous bariatric surgery, using appetite-regulating drugs and food allergies or intolerance. Periodic consultations were held with researchers and a nutritionist for 3 months. The initial and final evaluations involved the entire team, while monthly evaluations were only performed by the nutritionist. At the first visit, inclusion and exclusion criteria were investigated as well as the informed consent was obtained from all participants of the study.

We used the U.S. National Cholesterol Education Program's Adult Treatment Program III (NCEP-ATP III) [11] definition, which has been accepted by the First Brazilian Guidelines on the Diagnosis and Treatment of Metabolic Syndrome [12]. For a man being diagnosed with metabolic syndrome he has to have three or more of the following criteria: waist circumference > 102 cm, HDL-cholesterol < 40 mg/dL, triglycerides ≥ 150 mg/dL, fasting blood glucose ≥ 110 mg/dL or type 2 diabetes or blood pressure systolic ≥ 130 mmHg and/or diastolic ≥ 85 mmHg or hypertensive.

Hypogonadism symptoms were assessed using the Androgen Deficiency in the Aging Male (ADAM) questionnaire [13], while the Aging Male Symptoms (AMS) scale [14] was used to assess hormone deficiency symptoms. Erectile function was assessed using the International Index of the Erectile Function-5 (IIEF-5) [15].

Anthropometric measurements were performed according to World Health Organization norms [16]. All measurements were performed by the same investigator. All laboratory tests were performed at the Hospital de Clínicas de Porto Alegre. Participants with total serum testosterone < 300 ng/dL (chemiluminescence) and/or calculated free testosterone levels < 6.5 ng/dL, after two consecutive measures, were considered hypogonadal [17].

The control group was instructed to continue eating normally but received guidance about healthy eating patterns. The low-carbohydrate group was instructed by a nutritionist, member of the study team, to reduce carbohydrate intake and increase protein and fat intake [18]. Their diet could not contain more than 25–30% carbohydrates per day, aiming for 20–30 g carbohydrate per day. The two diets have the same amount of calories.

Data analysis was performed using SPSS Statistics (IBM, Armonk, NY, USA). Descriptive statistics (mean, standard deviation, frequency) were used. Normal distribution of the data was evaluated with Shapiro Wilks test. Student’s t test and Mann–Whitney U tests were used for normally distributed and non-normally distributed quantitative variables, respectively. In the comparison of qualitative data, Chi-Square and McNemar tests were used, respectively for dependent and independent samples. Pearson correlation analysis was used to examine the relationships between parameters that show normal distribution. *P*-values < 0.05 were considered statistically significant. Quantitative variables and analysis with Student's *t*-test were used to calculate the sample size. Using data from the literature, a standard deviation of 150 ng/dL for total serum testosterone, a detectable testosterone difference of 100 ng/dL, a significance level of 5%, and a power of 80% in a two-tailed test, 35 participants were required in each group. Considering a 20% loss, 44 participants were required per group, totaling 88 men. The datasets used and analyzed during the current study are available from the corresponding author on reasonable request.

The study was approved by the Research Ethics Committee of the Hospital de Clínicas de Porto Alegre (2017–0523). This study was carried out in accordance with the Declaration of Helsinki and Brazilian regulatory guidelines and norms for research involving human beings (National Health Council Resolutions 466/12 and 441/11). This study was registered at Plataforma Brasil (CAAE 61,385,716.8.0000.5327) and clinicaltrials.gov (NCT05019859, 25/08/2021).

## Results

A total of 22 men were included, 4 of whom dropped out after the first visit, 1 of the control group and 3 of the low carbohydrate one, due to personal problems. Thus 18 participants completed the study, ie, a 20% loss, which is common in clinical studies being 6 in the control group and 12 in the low carbohydrate group. Our study was conducted from March 2018 to October 2020.

The groups were homogeneous regarding the most relevant demographic data (Table 1). The mean age was 59.8 and 57 years in the control and low-carbohydrate groups, respectively (*p* = 0.46). The prevalence of both hypertension and diabetes was 57% in the sample, with no significant difference between the groups (hypertension: 43% vs 64%, *p* = 0.35; diabetes: 86% vs 43%, *p* = 0.06). A total of 19% of the participants were smokers, with no significant difference between the groups (29% vs 14%, *p* = 0.43). Regarding ethnicity, 71% self-reported as Caucasian, with no significant difference between the groups (86% vs 64%, *p* = 0.3). Erectile dysfunction was reported by 62%, which also did not differ between groups (86% vs 50%, *p* = 0.11).

**Table 1:** General characteristics of population

	**Control group** **(*n* = 6)**	**Low Carbohydrate group (*n* = 12)**	***P*-value**
Mean age (years)	59.8	57	**0.46**
White (%)	86	64	**0.30**
Smokers (%)	29	14	**0.43**
Diabetes mellitus (%)	86	43	**0.06**
Arterial hypertension (%)	43	64	**0.34**
Erectile dysfunction (%)	86	50	**0.11**
Weight (kg)	98.3	96.5	**0.87**
BMI (kg/cm2)	30.2	31.7	**0.31**
Total testosterone (ng/dL)	217.7	229.1	**0.63**
Calculated free testosterone (ng/dL)	4.3	4.7	**0.46**

Erectile function differed significantly between the groups, with a mean 2.4 points increase in the low-carbohydrate group at IIEF-5 questionnaire, which placed it in the mild erectile dysfunction category, rather than the mild-to-moderate category (15.5 points to 17.9 points, *p* < 0.01) (Table 2). The score also increased in the control group, although not enough to change its category, which remained mild-to-moderate (*p* = 0.3) (Table 2).

**Table 2:** AMS and IIEF-5 scores before and after the intervention

	**IIEF-5**		
	**Before**	**After**	***P*-value**
Control group (*n* = 6)	12	14.2	**0.3**
Low Carbohydrate group (*n* = 12)	15.5	17.9	**0.01**
	**AMS**		
Control group (*n* = 6)	41.8	44.7	**0.3**
Low Carbohydrate group (*n* = 12)	45.9	34.8	** < 0.01**

*AMS* Aging Male Symptoms scale, *IIEF-5* International Index of Erectile Function—5

Symptoms suggestive of hypogonadism significantly improved in the low-carbohydrate group according to AMS (*p* < 0.01) and ADAM (*p* < 0.01) scales. The AMS scores reduced by a mean of 11 points in the low-carbohydrate diet group, changing its symptom category from moderate to mild (Table 2). According to the ADAM questionnaire, the percentage of men with symptoms of hypogonadism in the low-carbohydrate group decreased from 78.6% before the intervention to 21.4% after the intervention, while in the control group, this percentage decreased from 100% to 85.7%. The difference in reduction between groups was significant (*p* < 0.01) (Table 3). If hypogonadism is considered by total testosterone serum level < 300 ng/dL, 100% was hypogonadal at the beginning of the study. However, the percentage of men who were eugonadal (total testosterone ≥ 300 ng/dL) at the end of intervention period was 3 times higher in the low-carbohydrate group (*p* = 0.05) (Table3).

**Table 3:** Hypogonadism and symptoms of hypogonadism prevalence using serum total testosterone level and ADAM score before and after the intervention

	**Control gourp** **(*n* = 6)**		**Low Carbohydrate group (*n* = 12)**		
	**Before**	**After**	**Before**	**After**	***P*-value**
Total testosterone < 300 ng/dL (%)	100	83.3	100	50	**0.05**
Symptoms of hypogonadism by ADAM score (%)	100	85.7	78.6	21.4	** < 0.01**

*ADAM* Androgen Deficiency in the Aging Male scale

Blood pressure levels were considered separately as systolic and diastolic. Systolic blood pressure was significantly reduced in the low-carbohydrate group but not in the control group (-9.07 mmHg vs—2.57 mmHg, *p* = 0.002). However, this difference did not occur in diastolic blood pressure (-3.64 mmHg vs + 3.85 mmHg, *p* = 0.12). Regarding anthropometric measurements, there was a significant weight reduction in both groups, although it was greater in the low-carbohydrate group (Table 4).

**Table 4:** End points before and after the intervention

	**Control gourp** **(*n* = 6)**			**Low Carbohydrate group** **(*n* = 12)**		
	**Before**	**After**	***P*-value**	**Before**	**After**	***P*-value**
Weight (Kg)	98.3	97.3	**0.04**	96.5	91.9	**0.002**
Body Mass Index	30.2	29.7	**0.07**	31.7	30.0	**0.01**
Abdominal circumference (cm) *	113.3	110.5	**0.06**	112.2	106.3	**0.002**
Hip circumference (cm)	105.7	105.2	**0.41**	104.3	102.3	**0.21**
Waist circumference (cm) **	108	107.3	**0.39**	106.4	102.8	**0.10**
Total testosterone (ng/dL)	217.7	227.2	**0.76**	229.1	310.7	**0.002**
Calculated free testosterone (ng/dL)	4.3	4.8	**0.58**	4.7	6.7	**0.001**

^*^Tape measure around person’s umbilical scar

^**^Tape measure around person’s middle (between the bottom of the ribs and the top of the hipbones)

The total serum testosterone increase was greater in the low-carbohydrate group than the control group (+ 81.6 ng/dL vs, + 9.5 ng/dL; *p* = 0.08). However, in the same group assessment (before-and-after model), there was a significant difference in the low-carbohydrate group but not in the control group (Table 4). Calculated free testosterone also differed in the low-carbohydrate group, as same group assessment, with a mean increase of 2 ng/dL (*p* = 0.001), resulting in a mean value of 6.7 ng/dL which is in the normal range. In the control group, the calculated free testosterone level did not differ significantly, with a mean increase of 0.45 ng/dL (*p* = 0.58) (Table 4).

## Discussion

Our findings were consistent with the results found in the literature as improving cardiovascular risk factors and anthropometric measurements as BMI but showed a possible improvement in sexual function and testosterone which was an unprecedented point related to a low-carbohydrate diet effect.

An excellent review, looked at studies along 40 years, found that men with metabolic syndrome and subnormal testosterone level should be advised to implement lifestyle measures such as weight loss and exercise, which, if successful, raise testosterone and provide multiple health benefits [19]. Another review of all well-conducted studies on low-carbohydrate diet, found reductions in weight and triglycerides in every population [20]. Along the same way, a meta-analysis compared a low-fat diet with a low-carbohydrate diet, finding that the latter was more effective at reducing weight and improving cholesterol and triglyceride levels [21].

Male sexual health and different diets were reviewed and some improvement was seen in men on Mediterranean diet [22]. We also found a sexual improvement in men after a low-carbohydrate diet, which is very similar to the Mediterranean diet. In 2021, Machado FP et al. [23] published a cohort study with 33 men assessing the hormonal profile and sexual function before and after bariatric surgery. Similar to our study, there was a significant increase in total testosterone and calculated free testosterone as well as a significant improvement in sexual function. However, unlike our study, these results were obtained after an invasive intervention such as bariatric surgery. Freedland SJ et al. [24] evaluated the effect of a low carbohydrate diet on cardiovascular and metabolic syndrome risk in men with prostate cancer. The authors found that this diet improved all parameters of metabolic syndrome, significantly reducing the chance of having it (OR 0.95; 95% CI 0.91–0.99; *p* = 0.023), corroborating the results of our study.

However, our study has some limitations. Since it is a diet, not all eligible patients agreed to participate. There is a great resistance to healthier diets, such as a low-carbohydrate diet, precisely because it affects entrenched eating habits. Nevertheless, the COVID-19 pandemic was another important limiting factor of our study, impeding the recruitment of new patients which led us to terminate the study prematurely even with a small sample size. The combination of these factors reduced the power of our results and limited our conclusions but it did not invalidate them and would encourage further researches on this topic.

## Conclusions

Our study demonstrated that a low-carbohydrate diet could reduce weight, systolic blood pressure and anthropometric measurements, especially abdominal circumference. Furthermore, a period of three months on this diet also raise the possibility of increasing serum levels of total and calculated free testosterone and of improving erectile function. However, larger studies should be done to demonstrate the potencial effect of a low-carbohydrate diet on serum testosterone level and erectile function in hypogonadal men with metabolic syndrome.

## Data Availability

The datasets used and analyzed during the current study are available from the corresponding author on reasonable request.

## References

[1] Brazilian Government database. https://www.gov.br/pt-br/noticias/saude-e-vigilancia-sanitaria/2020/10/pesquisa-do-ibge-mostra-aumento-da-obesidade-entre-adultos. Accessed 10 Jul 2022.

[2] Flegal KM, Carroll MD, Kit BK (2012). Prevalence of obesity and trends in the distribution of body mass index among US adults, 1999 and 2010. JAMA.

[3] Isidori AM, Buyat J, Corona G (2014). A critical analysis of the role of testosterone in erectile function: from pathophysiology to treatment - a systematic review. Eur Urol.

[4] Corona G, Forti G, Maggi M (2008). Why can patients with erectile dysfunction be considered lucky? The association with testosterone deficiency and metabolic syndrome. Aging Male.

[5] Stanworth R, Jones TH (2009). Testosterone in obesity, metabolic syndrome and type 2 diabetes. Front Horm Res.

[6] Rao PM, Kelly DM, Jones TH (2013). Testosterone and insulin resistance in the metabolic syndrome and T2DM in men. Nat Rev Endocrinol.

[7] Malik S, Wong ND (2009). Metabolic syndrome, cardiovascular risk and screening for subclinical atherosclerosis. Expert Rev Cardiovasc Ther.

[8] DeLay KJ, Haney N, Hellstrom WJ (2019). Modifying risk factors in the management of erectile dysfunction: a review. World J Men's Health.

[9] Hession M, Rolland C, Kulkami U (2009). Systemic review of randomized controlled trials of low-carbohydrate vs. low-fat/low-calorie diets in the management of obesity and its comorbidities. Obes Rev.

[10] Bazzano LA, Hu T, Reynolds K (2014). Effects of low-carbohydrate and low-fat diets: a randomized trial. Ann Inter Med.

[11] Executive Summary of the Third Report of The National Cholesterol Education Program (NCEP) Expert Panel on Detection, Evaluation, and Treatment of High Blood Cholesterol in Adults (Adult Treatment Panel III). JAMA. 2001;285(19):2486–97. 10.1001/jama.285.19.2486 11368702

[12] I Brazilian guidelines on diagnosis and treatment of metabolic syndrome. Arq Bras Cardiol. 2005;84 Suppl 1:1–28. 16095065

[13] Morley JE, Charlton E, Patrick P (2000). Validation of a screening questionnaire for androgen deficiency in aging males. Metabolism.

[14] Heinemann LA, Saad F, Heinemann K (2004). Can results of the Aging Males’ Symptoms (AMS) scale predict those of screening scales for androgen deficiency?. Aging Male.

[15] Rosen RC, Riley A, Wagner G (1997). The International Index of Erectile Function (IIEF): a multidimensional scale for assessment of erectile dysfunction. Urology.

[16] Physical status: the use and interpretation of anthropometry. Report of a WHO Expert Committee. World Health Organ Tech Rep Ser. 1995;854:1-452. 8594834

[17] Lunenfeld B, Mskhalaya G, Zitzmann M (2015). Recommendations on the diagnosis, treatment and monitoring of hypogonadism in men. Aging Male.

[18] Mavropoulos JC, Yancy WS, Hepburn J (2005). The effects of a low-carbohydrate, ketogenic diet on the polycystic ovary syndrome: a pilot study. Nutr Metabol.

[19] Grossmann M. Low testosterone in men with type 2 diabetes: significance and treatment. J Clin Endocrinol Metab. 2011;96(8):2341–53. 10.1210/jc.2011-0118 21646372

[20] Westman EC, Feinman RD, Mavropoulos JC (2007). Low-carbohydrate nutrition and metabolism. Am J Clin Nutr.

[21] Chawla S, Silva FT, Medeiros SA (2020). The effect of low-fat and low-carbohydrate diets on weight loss and lipid levels: a systematic review and meta-analysis. Nutrients.

[22] La J, Roberts NH, Yafi FA (2018). Diet and Men’s Sexual Health. Sex Med Rev.

[23] Machado FP, Rhoden EL, Pioner SR (2021). Weight loss through bariatric surgery in men presents beneficial effects on sexual function, symptoms of testosterone deficiency, and hormonal profile. Sex Med.

[24] Freedland SJ, Howard LE, Ngo A (2021). Low carbohydrate diets and estimated cardiovascular and metabolic syndrome risk in prostate cancer. J Urol.

